# Nrf2 mediates the effects of shionone on silica-induced pulmonary fibrosis

**DOI:** 10.1186/s13020-024-00947-5

**Published:** 2024-06-19

**Authors:** Guiyun Wang, Weixi Xie, Lang Deng, Xiaoting Huang, Mei Sun, Wei Liu, Siyuan Tang

**Affiliations:** 1 Shandong Xiehe University, Jinan, Shandong China; 2 Xiangya Nursing School, Central South University, Changsha, Hunan China

**Keywords:** Shionone, Nrf2, Oxidative stress, Myofibroblast differentiation, Macrophage activation, Silicosis

## Abstract

**Background:**

Extended contact with silica particles can lead to Silicosis, a chronic lung condition lacking established treatment protocols or clear mechanisms of development. The urgency for innovative treatments arises from the unavailability of effective treatment methodologies. The origin of silica-induced pulmonary fibrosis includes essential processes such as macrophage activation and the conversion of fibroblasts into myofibroblasts, with oxidative stress playing a pivotal role. Shionone (SHI), a triterpenoid extracted from the Aster tataricus plant, is recognized for its extensive health benefits. This study explores the capability of SHI to alleviate the effects of silica-induced lung fibrosis in mice.

**Methods:**

This investigation explored the impact of SHI on lung inflammation and fibrosis at different stages (early and late) triggered by silica in mice, focusing specifically on the initial and more developed phases. It comprised an analysis of isolated peritoneal macrophages and fibroblasts extracted from mice to elucidate SHI's therapeutic potential and its underlying mechanism. The methodology employed encompassed quantitative PCR, immunofluorescence, flow cytometry, and western blotting to examine macrophage activity and their transition into myofibroblasts. The activation of the nuclear factor erythroid 2-related factor 2 (Nrf2) pathway by SHI was confirmed via immunofluorescence and western blot studies. SHI's antioxidative properties were evidenced by the measurement of reactive oxygen species (ROS) and mitochondrial ROS within both macrophages and fibroblasts, using 2′, 7′-dichlorodihydrofluorescein diacetate and MitoSOX, respectively. The relevance of SHI was further underscored by applying ML385 and Nrf2 siRNA to gauge its effectiveness.

**Results:**

Starting SHI treatment early countered the harmful effects of lung inflammation and fibrosis caused by silica, while initiating SHI at a later phase decelerated the advancement of fibrosis. SHI's action was linked to the activation of the Nrf2 signaling pathway, a boost in antioxidant enzyme levels, and a decrease in oxidative stress and inflammation in macrophages affected by silica. Furthermore, SHI prevented the conversion of fibroblasts into myofibroblasts prompted by TGF-β, along with the resultant oxidative stress. The beneficial outcomes of SHI were negated when ML385 and Nrf2 siRNA were applied, highlighting the pivotal role of the Nrf2 pathway in SHI's efficacy.

**Conclusion:**

SHI plays a significant role in stimulating the Nrf2 pathway, thereby defending against silica-induced oxidative stress and inflammatory reactions in macrophages, and inhibiting the conversion of fibroblasts to myofibroblasts due to TGF-β. This suggests that SHI is a viable option for treating lung inflammation and fibrosis in mice suffering from silicosis.

**Supplementary Information:**

The online version contains supplementary material available at 10.1186/s13020-024-00947-5.

## Introduction

Exposure to silica (SiO2) dust over extended periods leads to Silicosis, a disease that primarily affects the lungs by causing persistent inflammation and the gradual thickening of lung tissue through fibrosis [1]. This condition evolves as immune cells invade the lungs, followed by the uncontrolled growth of fibroblast cells and an excessive buildup of the extracellular matrix (ECM) [2]. Despite advancements in workplace safety, the rates of Silicosis cases are on the rise worldwide, with a notable increase in less economically developed nations [3]. The precise biological processes contributing to Silicosis are yet to be fully understood, and the medical community lacks effective therapies to stop the disease's progression or reverse the damage in the lungs. Consequently, the creation of viable treatment options is critically needed.

Critical phases in the disease's progression include the activation of macrophages and the transformation of fibroblasts into myofibroblasts [4, 5]. When silica particles are inhaled, they trigger the activation of macrophages, which are key to the body's immune response. These cells gather in large numbers at inflammation sites and secrete a variety of substances that promote inflammation [6]. Persistent inflammatory signals lead fibroblasts to transition into myofibroblasts, which play a significant role in the development of fibrosis within the lungs [7]. By producing and releasing ECM substances, such as collagen, these myofibroblasts are instrumental in forming fibrotic areas within the lungs, deteriorating the organ's normal structure and function [8]. Thus, targeting macrophage activation and myofibroblast differentiation represents a promising approach to combating the effects of silica-induced pulmonary fibrosis.

Oxygen-reactive species, commonly abbreviated as ROS, encompass a variety of reactive molecules originating from oxygen that are produced both within organisms and in the natural surroundings. These entities are typically generated as a natural consequence of the body's oxygen use, where they play essential roles in cellular energy processes and communication pathways. Nevertheless, when exposed to harmful biological agents, the concentration of ROS can surge, inflicting significant harm on cell structures and their functionalities. This condition, known as oxidative stress, is noted by reference numbers [9, 10]. There's growing evidence suggesting oxidative stress is intricately linked to the control processes of lung fibrosis caused by silica, including the activation of macrophages and the transformation of myofibroblasts [6]. Addressing the modulation of oxidative stress in cases of fibrosis due to silicosis emerges as a pivotal approach for both prevention and treatment. To protect against damage induced by oxidative stress, our bodies deploy several antioxidant defense strategies. Central to these protective mechanisms is nuclear factor erythroid 2-related factor 2, also known as Nrf2, serving as a key regulatory factor [11, 12]. Under normal conditions, Nrf2 is attached to Keap-1, an acronym for Kelch-like ECH-associated protein 1. When oxidative stress occurs, Nrf2 detaches from Keap-1 [13, 14], migrates to the cell nucleus, and transforms into a transcription factor. This change sparks the activation of genes responsible for creating antioxidants and enzymes that detoxify, including heme oxygenase-1 (HO-1) and NAD(P)H quinone oxidoreductase-1 (NQO-1) [15]. Additionally, stimulating Nrf2 plays a crucial role in blocking macrophage activation and their transformation into myofibroblasts, underscoring the importance of Nrf2 as a target in treatment approaches for lung fibrosis resulting from silica exposure [16, 17].

Widely found across East Asia, the herb Aster tataricus is recognized for its beneficial impact on various respiratory conditions, including pharyngitis, cough, and asthma [18, 19]. Extracted from the plant's dried roots and rhizomes, Shionone (SHI) stands out as a triterpenoid with notable capabilities in modulating immune responses, reducing inflammation, and combating viruses [20, 21]. Despite its known benefits, the specific contributions and underlying mechanisms of SHI in addressing silica-induced pulmonary fibrosis remain unexplored. This research delves into how SHI influences silica-triggered pulmonary fibrosis, focusing on its interaction with macrophage activation and the transformation into myofibroblasts.

## Materials and methods

### Animals

Male C57BL/6 mice, aged eight weeks, were acquired and underwent adaptive feeding for a week at the Central South University Animal Center in Changsha, China. Following this period, anesthesia was administered to the mice, and their tracheas were subsequently injected with either silica (2.5 mg per mouse) or saline, marking the commencement of the experiment on day 0. The first batch of mice was administered SHI (12.5, 25, or 50 mg/kg) or saline by gavage over the next 14 consecutive days and sacrificed on days 14 or 48. The second batch of mice was administered SHI (12.5, 25, and 50 mg/kg) or saline by gavage from day 28 to day 42 and sacrificed on day 42. The mice in the first and second batches were randomly divided into five groups and treated as follows: (1) Control (CON) group, saline (intratracheal, *i.t.*) + saline (oral, *p.o*); (2) SiO_2_ group, silica + saline (*p.o*); (3) SiO_2_ + 12.5 mg/kg, silica + SHI (12.5 mg/kg, *p.o*); (4) SiO_2_ + 25 mg/kg, silica + SHI (25 mg/kg, *p.o*); (5) SiO_2_ + 50 mg/kg, silica + SHI (50 mg/kg, *p.o*). Six mice were in each group.

To observe the safety of SHI, mice were randomly divided into two groups and treated continuously for 14 days. The CON group received physiological saline (p.o*.*) and the SHI group received shionone (50 mg/kg, p.o*.*). Each group consisted of six mice.

SHI (National Institutes for Food and Drug Control, China, purity ≥ 99.5%) (Supplementary Fig. 1) was diluted in vehicle consisting of saline containing 5% (*v/v*) dimethyl sulfoxide (DMSO; provided by Shanghai Macklin Biochemical Co., Ltd., Shanghai, China). The methodology for all animal-based studies adhered strictly to the International Guidelines for Biomedical Research involving Animals.

### Histological evaluation

A comprehensive analysis was conducted on lung tissue using a grading system from 0 to 4, which incorporated five distinct factors: the extent of mixed cellular response within alveoli, the proliferation of bronchoalveolar structures, presence of bleeding, accumulation of lipoproteins within alveoli, and formation of hyaline membranes. This evaluation generated a cumulative lung damage score, independently determined by three pathologists who were not informed about the experimental data [22].

### Extraction and culture of primary peritoneal macrophages and primary mouse fibroblasts

To obtain primary peritoneal macrophages, mice were given a 3 mL intraperitoneal dose of 3% thioglycolate medium (Sigma-Aldrich, St. Louis, MO, USA). After allowing a period of four days for incubation, the mice then underwent a lavage using 15 mL of RPMI 1640 solution (Gibco, Waltham, MA, USA). The harvested cell blend was distributed evenly across culture dishes and then incubated at room temperature for 2 h.

For the isolation of primary lung fibroblasts, an approach using enzymatic cleavage was employed. The procedure began with the mice being anesthetized with sodium pentobarbital, followed by perfusion of their hearts with ice-cold PBS. The lungs were then excised, rinsed in cold PBS, and dissected into pieces measuring 1–2 cm^2. These sections were then subjected to enzymatic cleavage in a solution of DMEM containing 1 mg/mL collagenase I and incubated at 37 °C for an hour. Post-digestion, the mixture was strained through mesh filters of 70 and 40 μm, centrifuged, and the resulting sediment was resuspended in DMEM-high glucose (Gibco) enriched with 20% fetal bovine serum (FBS, Sigma-Aldrich) and 1% antibiotic–antimycotic solution. The fibroblasts were subsequently cultured at 37 °C in a moisture-saturated atmosphere containing 5% CO2.

In an environment where conditions were meticulously regulated, cellular cultures were sustained at a constant temperature of 37 ℃, under an atmospheric composition of 5% CO2. In detail, primary peritoneal macrophages were cultured in a medium enriched with 10% FBS (Gibco) and supplemented with a 1% antibiotic solution containing penicillin and streptomycin (Procell Life Science & Technology, Wuhan, China). In a parallel approach, primary mouse fibroblasts were nurtured in an environment that contained 20% FBS and an identical dosage of antibiotics. The study's methodology required subjecting certain cellular groups to various concentrations of transforming growth factor-beta 1 (TGF-β1, 10 ng/mL, PeproTech, Cranbury, NJ, USA) and SHI (in doses of 3, 10, or 30 μM) for a timeframe of 48 h.

### Levels of myeloperoxidase (MPO), malondialdehyde (MDA), superoxide dismutase (SOD), and glutathione (GSH)

The lung tissue was ground and homogenized to tested with Myeloperoxidase assay kit, Malondialdehyde (MDA) assay kit, Superoxide Dismutase (SOD) assay kit, glutathione (GSH) assay kit (Jiancheng Bioengineering Institute, China) according to the manufacturer's instructions.

### Measurement of the lung hydroxyproline

To measure the hydroxyproline content within lung tissues, we employed a method that made use of a Hydroxyproline Assay Kit, which was provided by the Nanjing Institute of Built-up Biotechnology in Nanjing, China. This approach required the processing of lung tissue samples (1 mg to 9 mL, weight/volume) with a digestion solution at a ratio of 5 to 1 (volume/volume), which were then incubated in a water bath set to maintain a steady temperature of 37 °C for a duration of 3 h. Following this step, an application solution was introduced to the specimens, which were then subjected to incubation at 60 °C within a water bath. The next phase involved centrifugation at 3500 rpm for a quarter-hour at a cold temperature setting of 4 °C. To determine the hydroxyproline concentration, we measured the optical density at a 550 nm wavelength.

### ROS and mitoROS assay

In the study focusing on reactive oxygen species (ROS) and mitochondrial ROS (MitoROS), analysis was conducted on primary macrophages and fibroblasts. The detection of ROS was carried out using 2′, 7′-dichlorodihydrofluorescein diacetate (H2DCFDA), acquired from Thermo Fisher Scientific. For assessing mitochondrial ROS, we applied the MitoSOX assay from the Nanjing Jiancheng Bioengineering Institute, located in Nanjing, China. Cells, after incubating for half an hour, were washed three times with PBS. This process allowed for the measurement of ROS and MitoROS levels through fluorescence microscopy with equipment provided by Nikon, headquartered in Tokyo, Japan.

### Flow cytometry

Regarding the assessment of ROS through flow cytometry, primary cells received a pretreatment with H2DAFDA for 30 min at 37 ℃. Following a thrice-repeated PBS wash, the cells were suspended anew for ROS level evaluation via flow cytometry, utilizing the LSR Fortessa system from BD, located in Santa Clara, CA, USA. Analysis of the collected data was facilitated through the employment of FlowJo version 10 software, developed by FlowJo in Eugene, OR, USA.

### Polarization of macrophage

Primary peritoneal macrophages were stained with allophycocyanin (APC) Anti-Mouse CD80 (Elabscience, Wuhan, China) and fluorescein isothiocyanate (FITC) Anti-Mouse CD206 (Elabscience) to determine the proportion of polarized macrophages. Flow cytometric analysis of the samples was carried out utilizing the LSR Fortessa device as previously mentioned. The acquired data underwent evaluation through the FlowJo software, version 10.

### Immunofluorescence

In the initial procedure, macrophages and fibroblasts were immobilized using a 4% paraformaldehyde solution for a quarter of an hour. Following this, the permeabilization of the cells was achieved by treating them with 0.5% Triton X-100 for 20 min, and thereafter, they were submerged in goat serum for half an hour. Overnight, at a temperature of 4 °C, the fibroblasts were incubated with specific antibodies targeting Nrf2 (obtained from Abcam, Cambridge, UK), P65 (procured from Proteintech, Rosemont, IL, USA), and alpha-smooth muscle actin (α-SMA; sourced from Proteintech). Post-incubation, a washing step with PBS was performed, after which the cells were exposed to either CoraLite488-conjugated Goat Anti-Rabbit IgG(H + L) or CoraLite594-conjugated Goat Anti-Rabbit IgG(H + L) (both supplied by Proteintech), and kept at 37 °C for an hour. Finally, the cells were examined under a fluorescence microscope equipped with a Zeiss ApoTome attachment (produced by Carl Zeiss, Oberkochen, Germany).

### Nuclear and cytoplasmic protein extraction

To begin the process of extracting nuclear and cytoplasmic proteins, the cells were first rinsed once with PBS before being dislodged with a cell scraper. This was followed by a centrifugation step to facilitate the separation of nuclear and cytoplasmic proteins, making use of a Nuclear Protein Extraction Kit from Solarbio (Beijing, China).

### Transfection with small interfering RNA (siRNA)

In the case of transfecting primary fibroblasts with small interfering RNA (siRNA), the procedure involved the use of siRNA designed to target Nrf2 along with a control siRNA, both sourced from Santa Cruz Biotechnology (Dallas, TX, USA). Lipofectamine RNAiMAX, a product of Invitrogen (Carlsbad, CA, USA), served as the vehicle for this transfection. The efficacy of the gene silencing was subsequently confirmed through western blot analysis.

### Western blotting

To isolate total proteins, lung tissues or cells underwent homogenization in RIPA buffer (Solarbio). The concentration of proteins was determined using the Pierce™ BCA Protein Assay Kit (Thermo Fisher Scientific). After this initial step, proteins with a 10% concentration were then subjected to separation via sodium dodecyl sulfate–polyacrylamide gel electrophoresis (SDS-PAGE) and subsequently transferred to polyvinylidene fluoride membranes (Bio-Rad, Hercules, CA, USA). In order to prevent non-specific binding, these membranes were initially soaked in a blocking solution consisting of 5% skim milk in PBS mixed with 0.1% Tween 20 (PBST). Following this, they were incubated at 4 °C overnight with primary antibodies directed against β-actin (1:5000 dilution, Proteintech), NF-κB P65 and its phosphorylated counterpart (1:1000 for each, obtained from Pho-NF-κB by Cell Signaling Technologies, Danvers, MA, USA), IκBα and its phosphorylated state (1:2500 and 1:10000 respectively, Abcam), HO-1 (1:1000, Proteintech), NQO-1 (1:1000, Proteintech), Collagen I (1:1000, Abcam), α-SMA (1:20000, Proteintech), and Lamin B1 (1:5000; Proteintech), NLR family pyrin domain containing 3 (NLRP3, 1:1000, Abcam), Apoptosis associated speck like protein containing CARD (ASC, 1:1000, Abclonal, Wuhan, China), Caspase-1 p10 (1:1000, Abcam), CD80 (1;1000, Cell Signaling Technology), CD206 (1:1000, Cell Signaling Technology), and Nrf2 (1:1000, Cell Signaling Technology). Following an in-depth wash with PBST, the samples were treated with horseradish peroxidase-tagged secondary antibodies for a two-hour period at ambient temperature. These antibodies included Rabbit Anti-Mouse IgG H&L (1:5000, Zen-Bio, Chengdu, China) and Goat Anti-Rabbit IgG H&L (1:5000, Zen-Bio). Subsequently, protein bands were detected utilizing Millipore's Luminata^™^ Crescendo chemi-luminescent horseradish peroxidase reagent, Burlington, VT, USA. The visual recording of these protein markers was achieved with a GeneGnome XRQ imager (Syngene, Cambridge, UK).

### Real-time quantitative PCR (qPCR) analysis

RNA was extracted from samples of lung tissue and cells utilizing TRIzol Reagent furnished by TransGen Biotech, located in Beijing, China. Post-extraction, the conversion to cDNA was carried out with a Reverse Transcription Kit procured from Novoprotein, based in Suzhou, China. Quantitative PCR analyses were conducted using SYBR GREEN, also supplied by Novoprotein, and carried out on a CFX96 Touch Real-Time PCR Detection System (Bio-Rad). The primers used in this process were acquired from Shanghai Sangong Biotechnology Co., Ltd., as specified in Table 1.

**Table 1:** Primer sequences for qPCR

Gene name	Forward	Reverse
*β-actin*	*CCTGCGACTTCAACAGCAAC*	*TGGGATAGGGCCTCTCTTGC*
*α-SMA*	*GCGTGGCTATTCCTTCGTGACTAC*	*CGTCAGGCAGTTCGTAGCTCTTC*
*Collagen I*	*GAGCGGAGAGTACTGGATCG*	*GCTTCTTTTCCTTGGGG-TTC*
*TNF-α*	*AGCCCCCAGTCTGTATCCTT*	*CTCCCTTTGCAGAACTCAGG*
*IL-6*	*CTGGGGATGTCTGTAGCTCA*	*CTGTGAAGTCTCCTCTCCGG*
*IL-1β*	*GGGCCTCAAAGGAAAGAATC*	*TACCAGTTGGGGAACTCTGC*
*HO-1*	*ACCGCCTTCCTCCTGCTCAACATTG*	*CTCTGACGAAGTGACGCCATCTG*
*NQO-1*	*GCGAGAAGAGCCCTGATTGTACTG*	*AGCCTCTACAGCAGCCTCCTTC*
*Nrf2*	*AAGCACAGCCAGCACATTCTCC*	*TGACCAGGACTCACGGGAACTTC*

### Respiratory function

Mice were anesthetized prior to tracheal intubation. Breathing frequency, pulmonary ventilation, lung compliance, and airway resistance of the mice were determined using the Max II system (Buxco Electronics, Inc., Wilmington, NC, USA).

### Statistical analyses

When analyzing the data, findings are presented as the average with the addition or subtraction of the standard deviation. This statistical evaluation was enabled through GraphPad Prism software (version 9.0), originating from San Diego, CA, USA. Initially, the Shapiro–Wilk test was applied to determine if the data distributions were normal. For datasets exhibiting a normal distribution, an analysis of variance (ANOVA) was undertaken to discern differences across multiple groups, with subsequent application of Tukey’s test for detailed comparisons between pairs. Conversely, the Kruskal–Wallis test was employed for datasets deviating from a normal distribution pattern. The examination of survival rates was performed utilizing the log-rank test, with a p-value less than 0.05 deemed indicative of statistical significance.

## Results

### Early-stage SHI treatment alleviates silica-induced pulmonary inflammation and fibrosis.

**Fig. 1:**
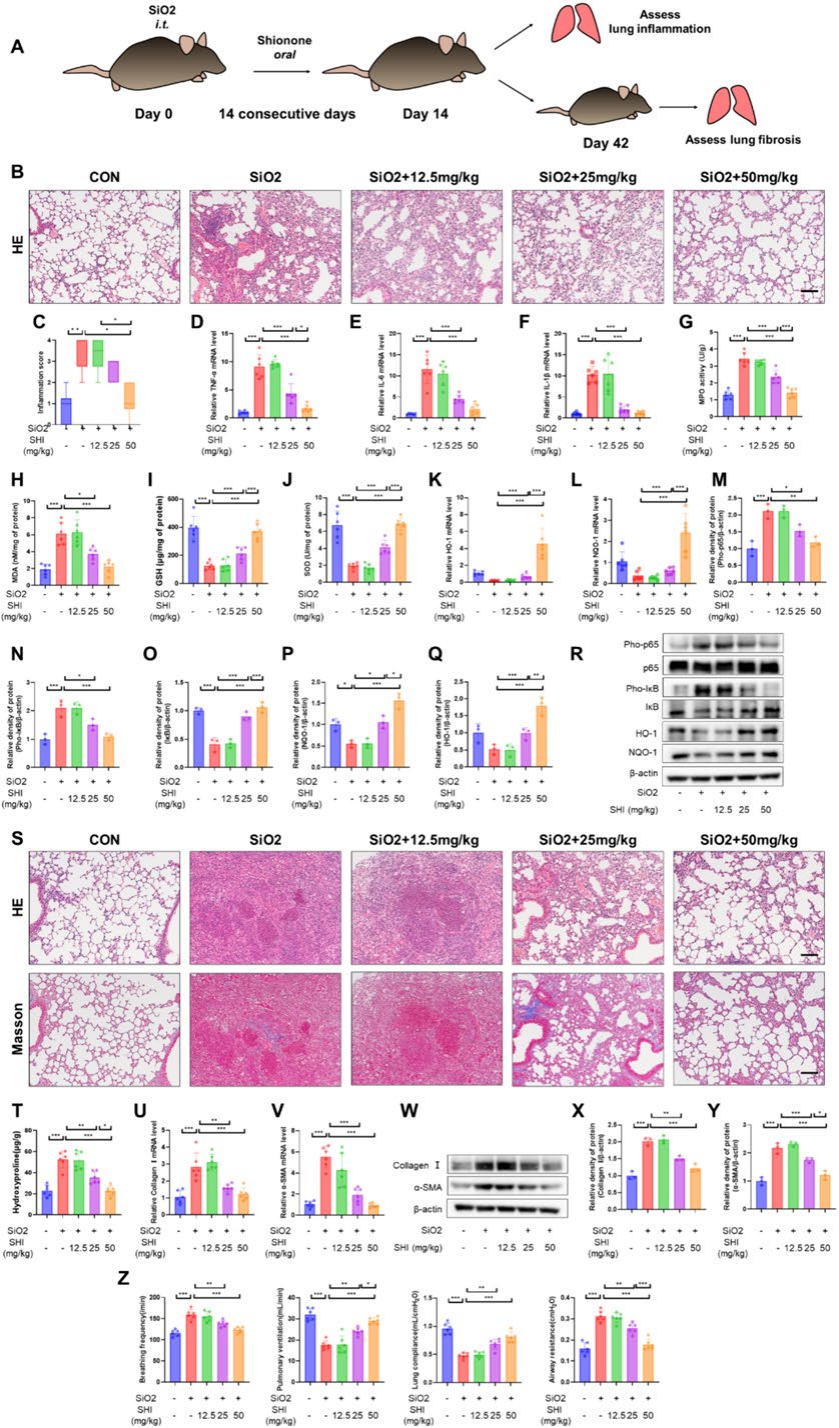
Early-stage SHI treatment alleviates silica-induced pulmonary inflammation and fibrosis. **A** Flow chart of animal model and SHI treatment. **B** Lung tissue HE staining after 14 days of silica treatment. **C** Lung inflammation score. **D**-**F** qPCR experiments to detect mRNA levels of TNF-α, IL-1β, and IL-6 in lung tissue. **G** Lung tissue MPO levels. **H**-**J** Biochemical assay to measure MDA content, GSH content, and SOD activity in lung tissue. **K**-**L** qPCR experiments to detect mRNA levels of HO-1 and NQO-1 in lung tissue. **M**-**R** WB experiments to measure the expression levels of Pho-p65, p65, Pho-IκB, IκB, HO-1, and NQO-1 in lung tissue. **S** Lung tissue HE staining and Masson staining after 42 days of silica treatment. **T** Biochemical assay to measure hydroxyproline levels in lung tissue. **U**-**V** qPCR experiments to detect mRNA levels of collagen I and α-SMA. **E**-**Y** WB experiments to measure the expression levels of collagen I and α-SMA in lung tissue. **Z** Breathing frequency, pulmonary ventilation, lung compliance, airway resistance. The scale bars represent 100 μm. Data are presented as means ± standard deviation (S.D), and all experiments were independently repeated at least three times. (*P < 0.05, **P < 0.01, and ***P < 0.001)

To assess the preliminary protective functions of SHI in countering silicosis, mice received oral administrations of SHI in dosages of 12.5, 25, and 50 mg/kg during the early stages of silica-induced lung inflammation (days 1–14). The objective was to scrutinize the impact of various SHI concentrations on pulmonary damage and the advancement towards fibrosis as depicted in (Fig. 1A). By day 14 following silica inhalation, pulmonary evaluations, HE staining, and inflammation scoring demonstrated a significant reduction in inflammatory cellular accumulation in the pulmonary regions of mice administered with SHI, in comparison to those without treatment (Fig. 1B, C). No death occurred in control or the SHI-treated groups of mice, and there were no significant differences in lung structure (Supplementary Fig. 2). SHI intervention counteracted the silica-induced activation of the NF-κB signaling pathway, as well as the upsurge in inflammatory factors and MPO levels (Fig. 1D-G, M–O, R). Given the role of oxidative stress in advancing silicosis, the study further investigated SHI's influence on oxidative stress triggered by silica. The treatment with SHI mitigated the rise in MDA levels, the decline in GSH levels, and the reduction in SOD activity instigated by silica (Fig. 1H–J). It also boosted the activity of antioxidant enzymes HO-1 and NQO-1 (Fig. 1K-L, P-R). On the 42nd day, the impact of initial SHI administration on fibrosis development was assessed. Analyses through HE and Masson’s trichrome staining, alongside hydroxyproline content determination, showed that SHI significantly lessened the severity of silica-driven pulmonary fibrosis in mice (Fig. 1S, T). It also overturned the indicators of fibrotic patches, including collagen I and α-SMA (Fig. 1U–Y), and notably enhanced respiratory functionality in the mice (Fig. 1Z). These outcomes indicate that initiating SHI treatment early can significantly protect against and ameliorate silica-induced lung damage and fibrosis in mice.

### Late-stage SHI treatment reduces silica-induced pulmonary fibrosis.

**Fig.2:**
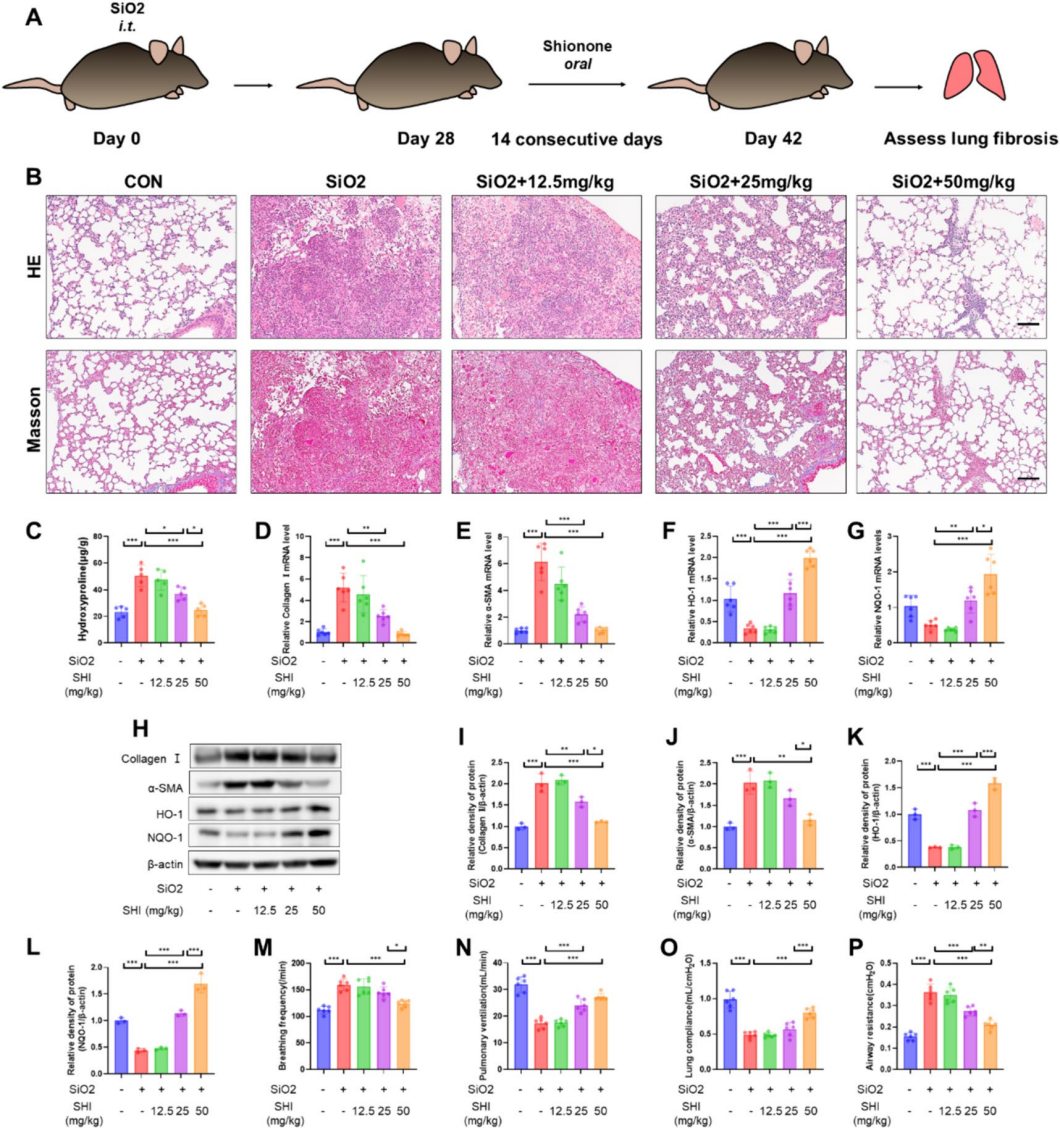
Late-stage SHI treatment attenuates silica-induced pulmonary fibrosis in mice. **A** Flow chart of animal model and SHI treatment. **B** Lung tissue HE and Masson staining after 42 days of silica treatment. **C** Biochemical assay to measure hydroxyproline levels in lung tissue. **D**-**G** qPCR experiments to detect mRNA levels of collagen I, α-SMA, HO-1 and NQO-1 in lung tissue. **H**–**L** WB experiments to measure the expression levels of collagen I, α-SMA, HO-1 and NQO-1 in lung tissue. **M**-**P** Breathing frequency, pulmonary ventilation, lung compliance, airway resistance. The scale bars represent 100 μm. Data are presented as means ± standard deviation (S.D), and all experiments were independently repeated at least three times. (*P < 0.05, **P < 0.01, and ***P < 0.001)

To analyze the direct anti-fibrotic properties of SHI, we pinpointed the essential timeframe for the development of fibrotic foci in silicosis (days 28–42) as the optimal period for administering SHI (Fig. 2A). Results from HE and Masson staining demonstrated that SHI ameliorated fibrotic foci formation in a dose-dependent fashion (Fig. 2B). Moreover, SHI diminished the increased hydroxyproline levels brought about by silica exposure, as well as the quantities of collagen I and α-SMA (Fig. 2C-E, H-J). SHI enhanced the expression of the antioxidant enzymes HO-1 and NQO-1 (Fig. 2F-H, K-L). Additionally, SHI improved the compromised respiratory capabilities caused by silica in mice (Fig. 2M–P). These findings indicate that administering SHI during the later stages effectively mitigates silica-triggered pulmonary fibrosis in mice.

### SHI mitigates silica-induced oxidative stress in macrophages and TGF-β1-induced oxidative stress in fibroblasts

**Fig. 3:**
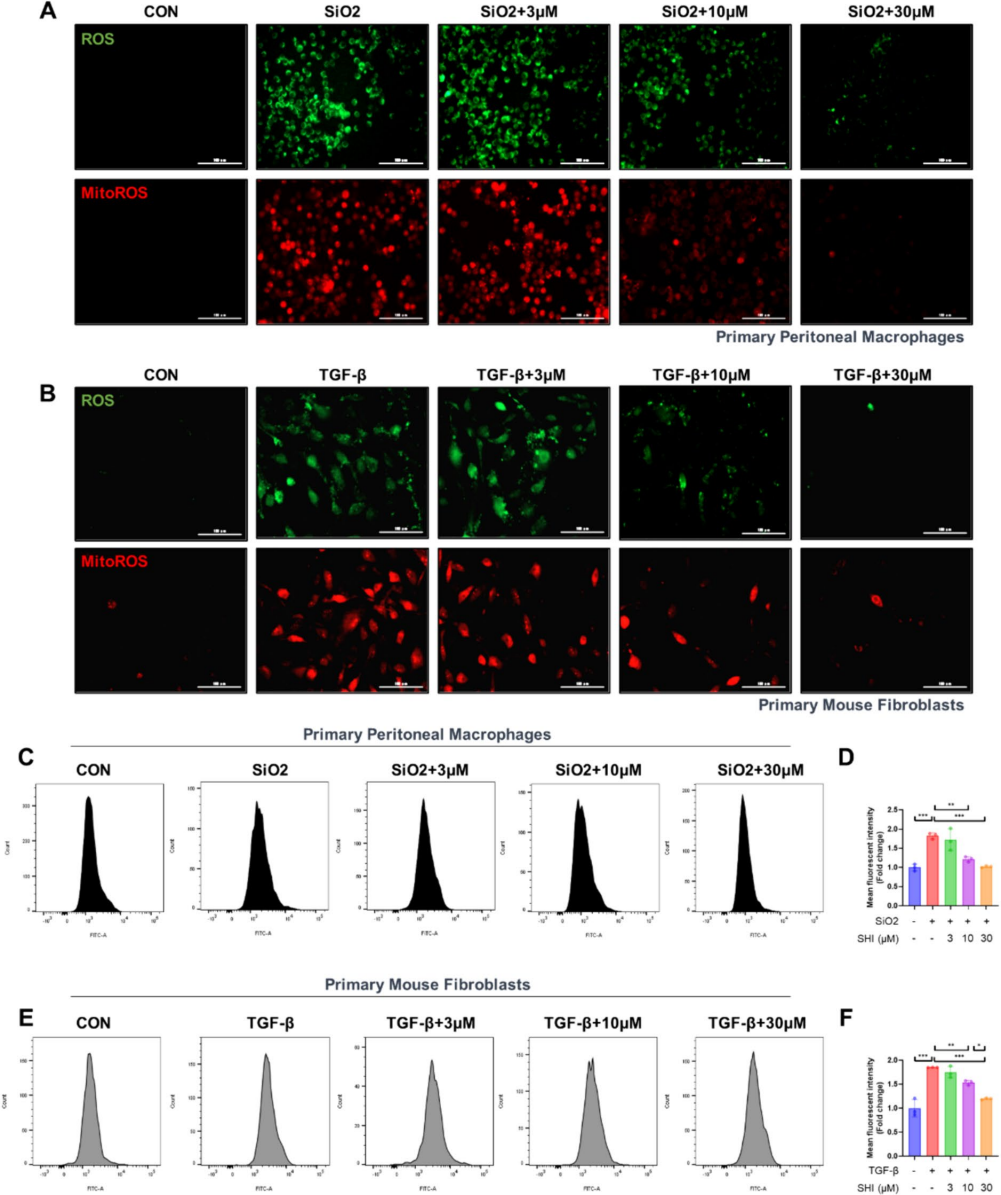
SHI reduces oxidative stress in silica-induced macrophages and TGF-β1-induced fibroblasts. **A** H2DCFCDA probe and MitoSOX probe labeling of ROS and MitoROS levels in primary peritoneal macrophages. (Green represents ROS, red represents MitoROS) (**B**) H2DCFCDA probe and MitoSOX probe labeling of ROS and MitoROS levels in primary mouse lung fibroblasts. (Green represents ROS, red represents MitoROS) (**C**-**D**) Flow cytometry detection of ROS levels in macrophages. **E**–**F** Flow cytometry detection of ROS levels in fibroblasts. The scale bars represent 100 μm. Data are presented as means ± standard deviation (S.D), and all experiments were independently repeated at least three times. (*P < 0.05, **P < 0.01, and ***P < 0.001)

In the exploration of SHI's capacity to counteract silica-induced oxidative stress within macrophages and the stress in fibroblasts caused by TGF-β1, we focused on the primary effector cells identified in various phases of silicosis. SHI was found to reduce the elevated ROS and mitoROS levels in primary peritoneal macrophages due to silica (Fig. 3A–D) and manifested antioxidant properties in primary fibroblasts challenged with TGF-β1 (Fig. 3B–F). These observations underline the significance of SHI's antioxidant effect in the context of silicosis, impacting both macrophages and fibroblasts.

### SHI activates the Nrf2 signaling pathway in macrophages and fibroblasts.

**Fig. 4:**
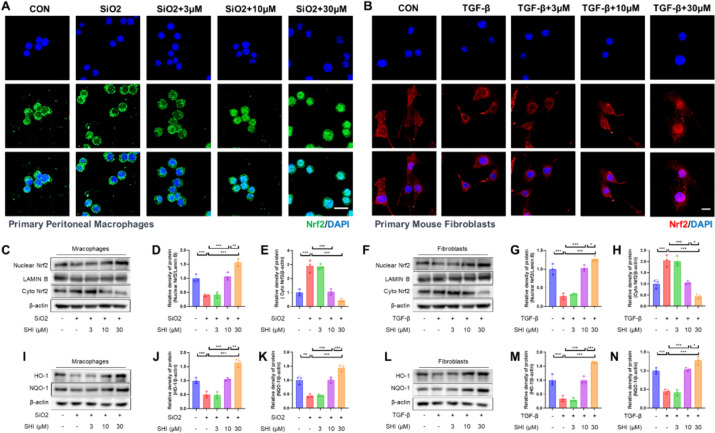
SHI activates Nrf2 signaling pathway in macrophages and fibroblasts. **A** IF assay detecting Nrf2 nuclear translocation in macrophages after 3 h of SHI treatment. **B** IF assay detecting Nrf2 nuclear translocation in fibroblasts after 3 h of SHI treatment. **C**-**E** Western blot experiment detecting Nrf2 levels in the nuclear and cytoplasmic proteins of macrophages. **F**–**H** Western blot experiment detecting Nrf2 levels in the nuclear and cytoplasmic proteins of fibroblasts. **I**-**K** Western blot experiment detecting the expression of HO-1 and NQO-1 in macrophages. **L**-**N** Western blot experiment detecting the expression of HO-1 and NQO-1 in fibroblasts. The scale bars represent 20 μm. Data are presented as means ± standard deviation (S.D), and all experiments were independently repeated at least three times. (*P < 0.05, **P < 0.01, and ***P < 0.001)

Delving into the Nrf2 signaling pathway, crucial for defense against oxidative damage [23], we assessed SHI's influence on this pathway. Analyses through immunofluorescence and western blot techniques revealed that SHI facilitated Nrf2's nuclear translocation within both macrophages and fibroblasts (Fig. 4A–H). Additionally, an increase in the activity of antioxidant enzymes HO-1 and NQO-1 was noted following SHI application (Fig. 4I–N). This evidence supports the proposition that SHI activates the Nrf2 signaling pathway across both cell types.

### SHI alleviates silica-induced macrophage inflammation activation.

**Fig. 5:**
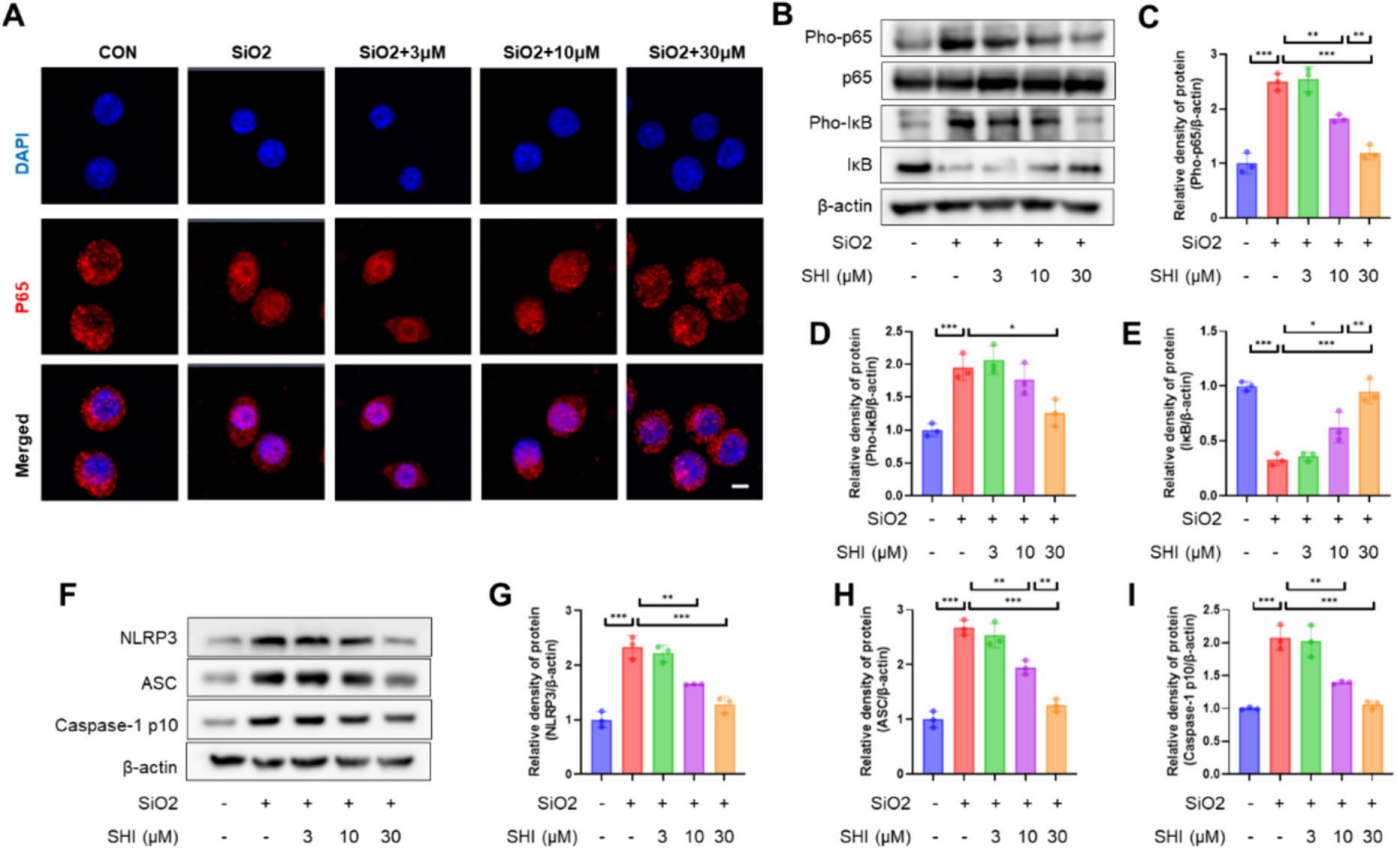
SHI reduces silica-induced macrophage activation and promoted M2 polarization. **A** After 3 h of SHI treatment, IF assay was conducted to detect the nuclear localization of p65 in macrophages. **B**-**E** WB assays was performed to assess the expression levels of Pho-p65, p65, Pho-IκB, and IκB in macrophages. **F**-**I** WB assays was performed to assess the expression levels of NLRP3, ASC and Caspase-1 p10 in macrophages. The scale bars represent 20 μm. Data are presented as means ± standard deviation (S.D), and all experiments were independently repeated at least three times. (*P < 0.05, **P < 0.01, and ***P < 0.001)

To evaluate the impact of SHI on the NF-κB pathway more thoroughly, we conducted studies using immunofluorescence and western blot techniques. Findings from the immunofluorescence examination revealed that SHI intervention disrupted the nuclear presence of p65, highlighting its influence on the NF-κB pathway (Fig. 5A). Analysis through western blot further substantiated SHI's role in reversing the activation within the NF-κB pathway, as evidenced by alterations in the levels of p65, Pho-p65, Pho-IκB, and IκB (Fig. 5B–E). Additionally, the interplay between the inflammasome's formation and activation, crucial for macrophage activation, and the NF-κB pathway intensifies the inflammatory response [24]. Evidence showed SHI's capability to dampen the inflammasome pathway (Fig. 1F–I). Moreover, SHI's influence on macrophage polarization was noted; it mitigated the shift of M0 macrophages to the M1 phenotype induced by silica, while fostering a shift towards the M2 phenotype (Supplementary Fig. 3A-D), indicating that SHI mitigates inflammation induced in macrophages by silica.

### Nrf2 mediates the anti-inflammatory and antioxidative effects of SHI on macrophages.

**Fig. 6:**
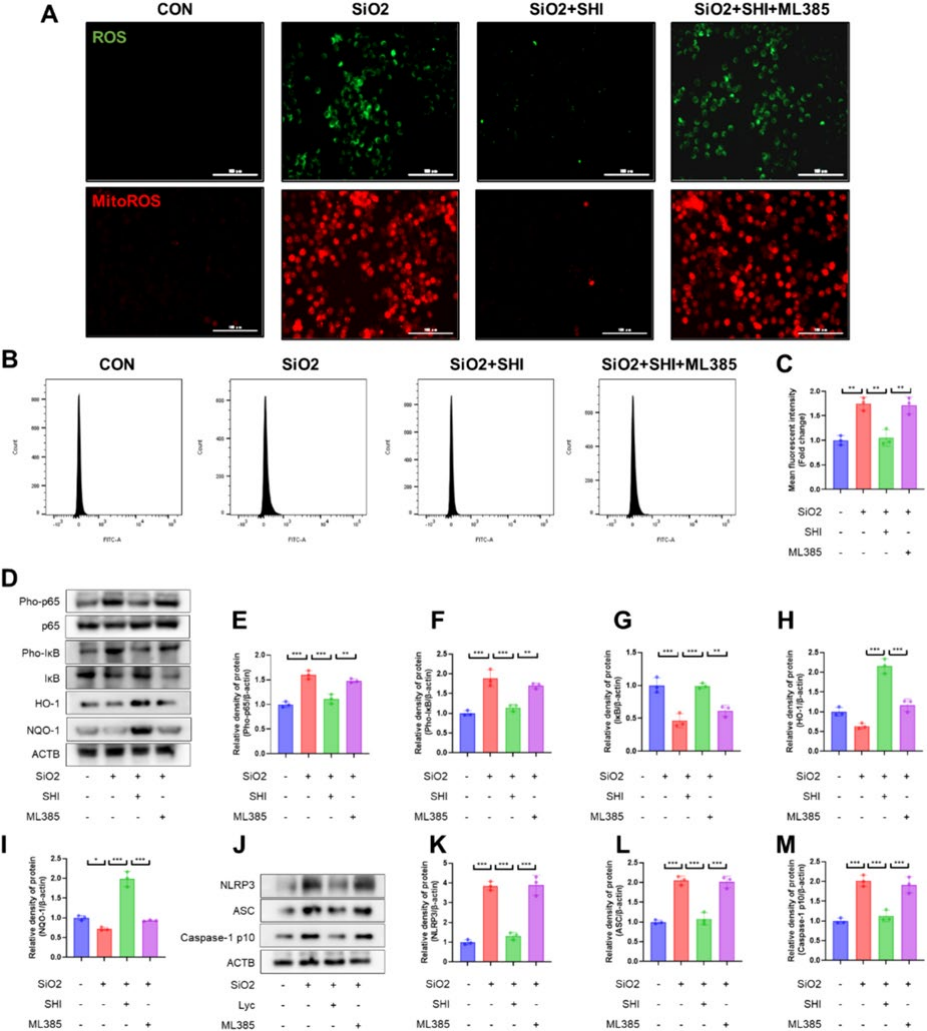
Nrf2 mediates the effects of SHI on macrophages. **A** H2DCFCDA probe and MitoSOX probe labeling of ROS and MitoROS levels in primary peritoneal macrophages. (Green represents ROS, red represents MitoROS) (**B**-**C**) Flow cytometry detection of ROS levels in macrophages. **D**-**I** WB assays was performed to assess the expression levels of Pho-p65, p65, Pho-IκB, IκB, HO-1 and NQO-1 in macrophages. **J**-**M** WB assays was performed to assess the expression levels of NLRP3, ASC and Caspase-1 p10 in macrophages. The scale bars represent 100 μm. Data are presented as means ± standard deviation (S.D), and all experiments were independently repeated at least three times. (*P < 0.05, **P < 0.01, and ***P < 0.001)

Investigations were carried out to understand if Nrf2 is responsible for the beneficial effects of SHI on macrophages, employing ML385, a specific Nrf2 inhibitor. ML385 negated SHI's beneficial impacts on ROS and mitoROS in macrophages (Fig. 6A–C). Moreover, western blot analyses showed that ML385 counteracted SHI's anti-inflammatory and antioxidative stress benefits in macrophages (Fig. 6D–M). ML385 also nullified SHI's positive effect on the polarization of macrophages (Supplementary Fig. 3E-H), suggesting Nrf2's pivotal role in SHI's anti-inflammatory and antioxidative influence on macrophages.

### SHI reduces TGF-β1-induced myofibroblast differentiation.

**Fig. 7:**
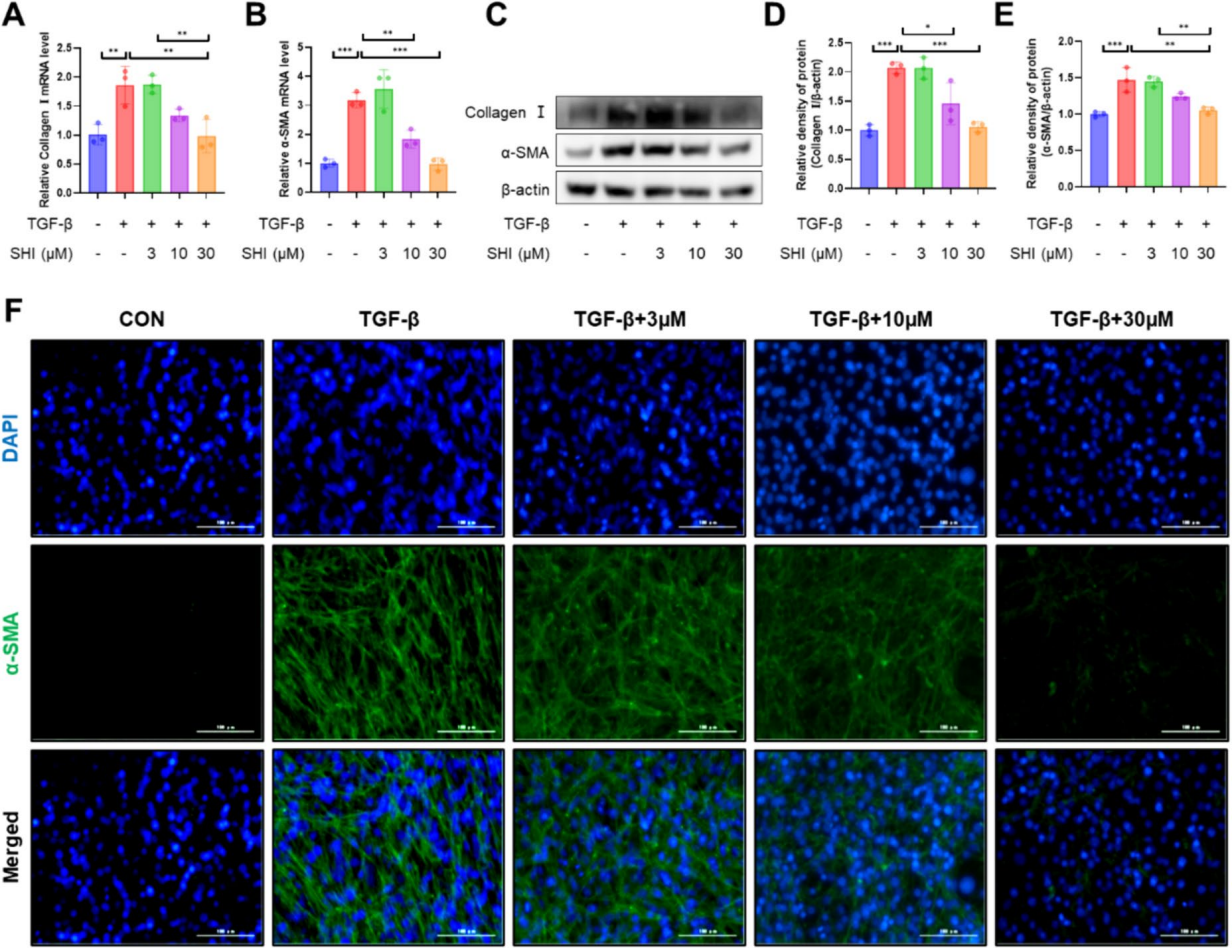
SHI reduces TGF-β1-induced myofibroblast differentiation. **A**-**B** qPCR experiments to detect mRNA levels of collagen I and α-SMA. **C**-**E** WB experiments to measure the expression levels of collagen I and α-SMA. **F** IF experiments to detect the expression levels of α-SMA. The scale bars represent 100 μm. Data are presented as means ± standard deviation (S.D), and all experiments were independently repeated at least three times. (*P < 0.05, **P < 0.01, and ***P < 0.001)

Myofibroblasts along with the extracellular matrix (ECM) constitute the primary elements of fibrotic areas, employing collagen and α-SMA as their biomarkers. The analysis through qPCR and western blotting has indicated a decrease in collagen I and α-SMA levels that is dependent on the concentration following the application of SHI (Fig. 7A–E). The data from immunofluorescence tests have shown a reduction in the TGF-β1-induced elevation of α-SMA due to SHI administration (Fig. 7F). This evidence supports the conclusion that SHI plays a role in obstructing the differentiation of myofibroblasts triggered by TGF-β1.

### Nrf2 mediates the anti-fibrotic and anti-oxidative effects of SHI on fibroblasts

**Fig. 8:**
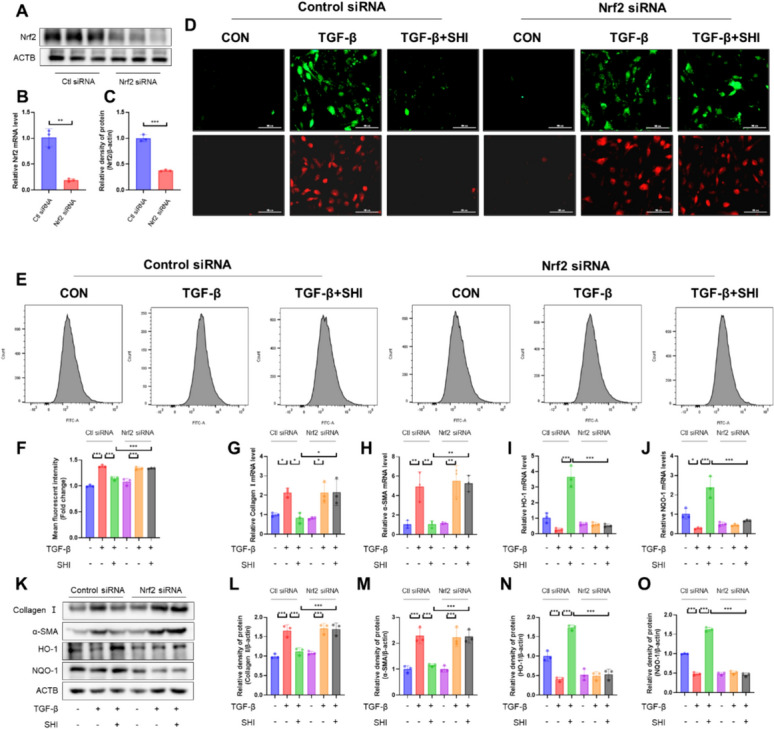
Nrf2 mediates the effects of SHI on myofibroblast differentiation. **A** qPCR experiments to detect mRNA levels of Nrf2. **B**-**C** WB experiments to measure the expression levels of Nrf2. **D** H2DCFCDA probe and MitoSOX probe labeling of ROS and MitoROS levels in primary mouse lung fibroblasts. (Green represents ROS, red represents MitoROS). **E**–**F** Flow cytometry detection of ROS levels in fibroblasts. **G**-**J** qPCR experiments to detect mRNA levels of HO-1, NQO-1, collagen I and α-SMA. **K**–**O** WB experiments to measure the expression levels of HO-1, NQO-1, collagen I and α-SMA. The scale bars represent 100 μm. Data are presented as means ± standard deviation (S.D), and all experiments were independently repeated at least three times. (*P < 0.05, **P < 0.01, and ***P < 0.001)

To confirm whether Nrf2 mediates the effects of SHI on fibroblast transformation, we established a model of Nrf2 knockdown in fibroblasts and validated the results using qPCR and western blot (Fig. 8A–C). The silencing of Nrf2 via siRNA counteracted SHI's suppression of ROS and mitoROS in fibroblasts (Fig. 8D–F). Furthermore, analyses via qPCR and western blotting illustrated that Nrf2 siRNA negated the influence of SHI on the differentiation of myofibroblasts and the modulation of antioxidant enzymes (Fig. 8G–O). This body of work proposes that Nrf2 is integral in the conveyance of SHI's protective effects against fibrosis and oxidative harm in fibroblasts.

## Discussion

This research marks a pioneering endeavor in demonstrating SHI's capability to engage Nrf2 activation, contributing to its defensive stance against silica-triggered pulmonary fibrosis and oxidative stress within the lungs, both in vitro and in vivo settings. The in vivo analysis highlighted SHI's dynamic benefits in mitigating fibrosis advancement through the suppression of initial inflammation and oxidative stress, alongside a direct anti-fibrotic action. Moreover, the in vitro results introduce groundbreaking evidence of Nrf2's involvement in SHI's defense against silica-induced activation of macrophages and the transformation of TGF-β1-stimulated myofibroblasts. Given these findings, SHI's inhibitory influence on key effector cells responsible for silica-related fibrosis positions it as an effective candidate for both preventing and treating silica-driven lung fibrosis.

Addressing silicosis involves strategies to halt disease progression, mitigate inflammation, curb fibrosis, and enhance overall well-being [25]. During the initial phase (days 1–14) following the tracheal introduction of silica particles, macrophages serve as the key cells in action, undergoing activation and polarization upon silica absorption. This process triggers the release of a plethora of factors that contribute to inflammation and fibrosis [26, 27]. As the condition progresses to days 28–42, fibroblasts ascend as pivotal cells, driving the conversion of fibroblasts into myofibroblasts influenced predominantly by TGF-β1. This activity results in the secretion of extracellular matrix components, culminating in the formation of fibrotic areas [2, 28–30]. Hence, this research endeavors to evaluate the preventative and curative capabilities of SHI against silicosis-induced lung fibrosis in murine models. Observations indicate that administering SHI orally at the early stages (days 1–14) diminishes the lung's inflammatory response and oxidative stress prompted by silica exposure, thereby forestalling fibrosis. Administering SHI orally in the latter stages (days 28–42) directly combats fibrosis. Additionally, SHI has been shown in vitro to mitigate the activation of primary peritoneal macrophages by silica and inhibit the TGF-β1 driven transformation of primary mouse fibroblasts into myofibroblasts. These insights suggest SHI's potential as an effective intervention for silicosis prevention and therapy.

Oxidative stress and inflammatory responses play crucial roles in silicosis [31]. Oxidative stress and inflammation are closely associated with various pathological processes. Under stimulating factors, macrophages generate a significant amount of ROS, RNS, inducing oxidative stress. Similarly, oxidative stress can induce inflammation via various pathways [32, 33]. For example, H_2_O_2_ can activate the NF-κB and inflammasome signaling pathways [34]. Furthermore, oxidative stress can amplify the inflammatory response, which promotes the expression of inflammatory factors. In turn, inflammatory cells can induce oxidative stress reactions by infiltrating lung tissue [35]. In this study, SHI treatment alleviated macrophage activation through the NF-κB and inflammasome signaling pathways. Furthermore, SHI reduced silica-induced M1 macrophage polarization and promoted M2 macrophage polarization. M2 macrophages play dual roles in the progression of lung fibrosis [36]. These macrophages alleviate early fibrosis-associated inflammation by reducing the secretion of inflammatory factors and secrete a large amount of cytokines, such as TGF-β and platelet-derived growth factor, to promote damaged epithelial repair and alleviate fibrosis [37]. However, cytokines like TGF-β can accelerate fibrosis formation by promoting fibroblast activation [38]. Therefore, to determine whether SHI can counteract the activation of M2 macrophages induced by silica in fibroblasts, we used conditioned media from SHI-treated macrophages or IL-4-treated macrophages to culture fibroblasts and observed fibroblast activation, migration, and invasion in the presence or absence of SHI. These results suggest that SHI inhibits the effect of M2 macrophages on fibroblasts (Supplementary Fig. 4), which may contribute to its overall anti-fibrotic effect. Several investigations have highlighted SHI's potential as an antioxidant and anti-inflammatory agent, citing its ability to counteract oxidative stress and inflammation [39, 40]. Laboratory tests reveal that administering SHI promptly can negate the alterations caused by silica in MDA, GSH, SOD levels, and inflammatory injuries. This suggests SHI's effectiveness in preventing or treating silicosis through its antioxidant and anti-inflammatory actions.

Research underscores the critical impact of oxidative stress in the development of silica-related lung scarring. The secretion of TGF-β1, essential for the transition of fibroblasts into myofibroblasts, is influenced by oxidative stress [41, 42]. Antioxidants play a pivotal role in managing lung scarring, as demonstrated by N-acetylcysteine's ability to prevent the transformation of fibroblasts in such conditions [43]. Our research focused on assessing the late-stage intervention of SHI in a murine model of silica-driven lung fibrosis. In these experiments, SHI treatment led to a decrease in MDA, an increase in GSH, and an improvement in SOD activity when administered. These outcomes hint at SHI's direct anti-scarring effects, possibly through its antioxidative capabilities. Moreover, SHI showed a reduction in both ROS and mitochondrial ROS generation during the process of myofibroblast transformation in lab conditions. Given that myofibroblasts are key players in ROS production, and their ROS output can trigger apoptosis in epithelial cells [44], SHI's role in mitigating ROS-induced apoptosis in epithelial cells highlights a promising anti-scarring strategy, meriting further exploration.

Nrf2 occupies a pivotal position in the cell's arsenal against oxidative harm, orchestrating the regulation of several antioxidant proteins [45]. The activation of the Nrf2 pathway is crucial for the prevention of silicosis [46]. Tanshinone IIA has been shown to stimulate this pathway, offering protection against silica-triggered lung fibrosis in rat models [47]. Additionally, a combined treatment involving N-acetylcysteine and desipramine has been found effective in reducing lung fibrosis caused by silica in rats, through the modulation of Nrf2 expression [48]. Laboratory findings indicate that SHI treatment encourages the movement of Nrf2 into the nuclei of macrophages and fibroblasts, leading to an increase in HO-1 and NQO-1 levels. Moreover, animal studies have validated that SHI triggers the Nrf2 pathway. The impact of SHI on these cells can be negated by blocking Nrf2, either with a targeted inhibitor or Nrf2 siRNA, showcasing Nrf2's role in SHI's capacity to counteract silica-driven lung fibrosis.

**Fig. 9:**
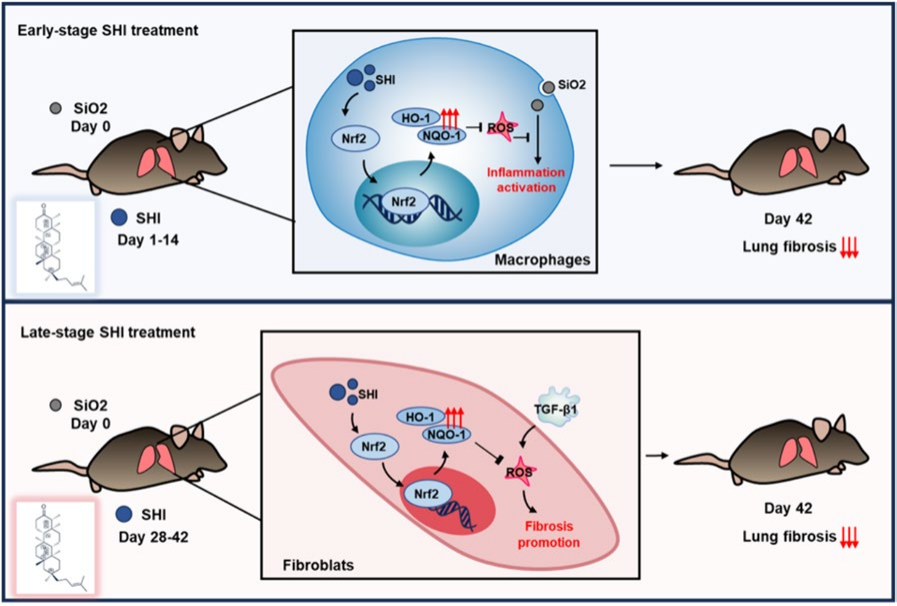
Schematic diagram of the proposed therapeutic mechanism of SHI for silica-induced fibrosis. In silica-induced fibrosis mice, early-stage SHI treatment (days 1–14), primarily targeting macrophages, promotes Nrf2 nuclear translocation and the expression of antioxidant enzymes, reducing ROS-mediated inflammatory activation and consequently alleviating lung inflammation and fibrotic progression. Late-stage SHI treatment (days 28–42), focusing on fibroblasts, reverses TGF-β1-induced myofibroblast differentiation regulated by ROS through the activation of the Nrf2 signaling pathway, thereby exerting antifibrotic effects

Our investigation sheds light on the potential role of SHI as a promising candidate for treating lung fibrosis induced by silicosis. Targeting Nrf2 with SHI demonstrates therapeutic effects on cells involved in various phases of silicosis. In macrophages, SHI's activation of the Nrf2 pathway mitigates the oxidative stress and inflammation prompted by silica. Similarly, in fibroblasts, SHI thwarts TGF-β1-driven transformation into myofibroblasts and oxidative stress via the Nrf2 pathway. The influence of SHI on these critical cell types encourages further research into its utility for treating other conditions linked to macrophages and myofibroblasts. This finding is a crucial advancement in creating innovative treatments for lung fibrosis stemming from silicosis (Fig. 9).

### Supplementary Information

Supplementary material 1: Fig. S1 The H-NMR and C-NMR spectra of SHI. (A) The 1H-NMR spectra copy of Shionone in CDCl3 (400 MHz). (B) The 13C-NMR spectra copy of Shionone in CDCl3 (101 MHz).

Supplementary material 2: Fig. S2 The effects of SHI treatment on pulmonary structure. Lung tissue HE staining and Masson staining after 14 days of SHI treatment.

Supplementary material 3: Fig. S3 The effects of SHI on macrophage polarization. (A) Flow cytometry was employed to detect macrophage polarization. APC-F4/80 was used to detect M1 polarization, while FITC-CD206 was utilized for detecting M2 polarization. (B-D) Western blotting was used to detect macrophage polarization. (E-G) Under Nrf2 treatment, Western blotting was employed to detect macrophage polarization. (H) Under Nrf2 treatment, Flow cytometry was employed to detect macrophage polarization. Data are presented as means ± standard deviation (S.D), and all experiments were independently repeated at least three times. (*P < 0.05, **P < 0.01, and ***P < 0.001).

Supplementary material 4: Fig. S4 The inhibitory effect of SHI on M2 macrophage-mediated activation of fibroblasts. Macrophages were treated with IL-4/Silica+SHI, and conditioned medium was obtained. The activation of fibroblasts was observed in the presence or absence of SHI. (A-B) qPCR experiments to detect mRNA levels of collagen I and α-SMA. (C-E) WB experiments to measure the expression levels of collagen I and α-SMA. (F) Scratch assay. The scale bars represent 1000 μm. Data are presented as means ± standard deviation (S.D), and all experiments were independently repeated at least three times. (*P < 0.05, **P < 0.01, and ***P < 0.001).

## Data Availability

The data used to support the findings of this study are available from the corresponding author upon request.

## Abbreviations

SHI: Shionone
TGF-β: Transforming growth factor-beta
ECM: Extracellular matrix
Nrf2: Nuclear Factor Erythroid 2-Related Factor 2
DMSO: Dimethyl sulfoxide
i.t*.*: Intratracheal
α-SMA: Alpha-smooth muscle actin
ROS: Reactive oxygen species
