# Supplementary figures and images for: Nrf2 mediates the effects of shionone on silica-induced pulmonary fibrosis
Source: Chin Med. 2024 Jun 19;19:88. doi: 10.1186/s13020-024-00947-5 (PMC11188511; doi:10.1186/s13020-024-00947-5)

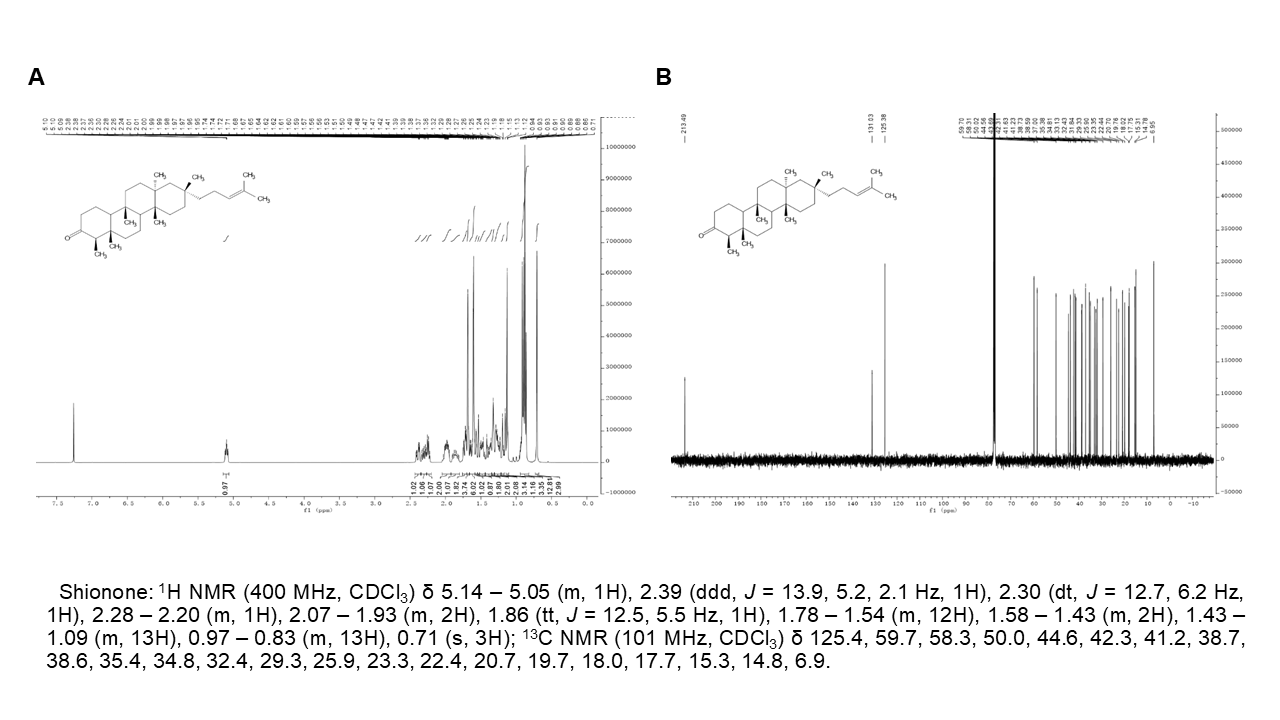

Supplement: Supplementary file 1 — Supplementary material 1: Fig. S1 The H-NMR and C-NMR spectra of SHI. (A) The 1H-NMR spectra copy of Shionone in CDCl3 (400 MHz). (B) The 13C-NMR spectra copy of Shionone in CDCl3 (101 MHz). [file 13020_2024_947_MOESM1_ESM.tif]

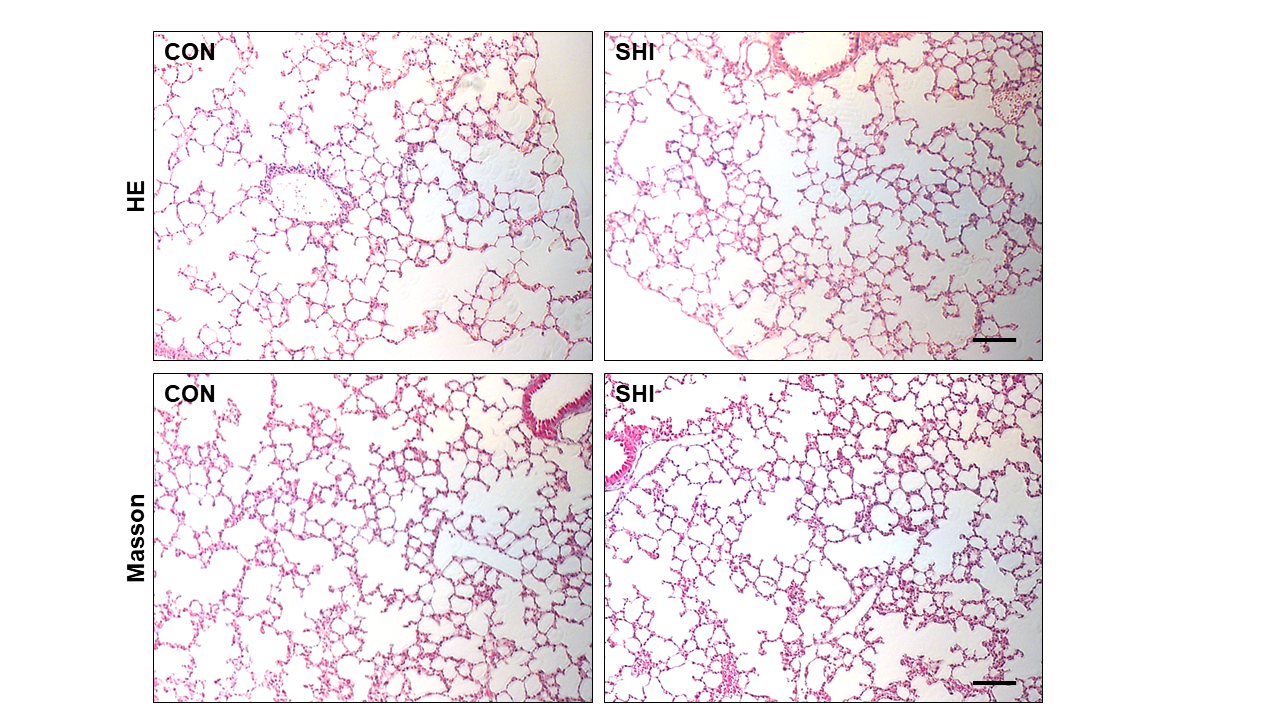

Supplement: Supplementary file 2 — Supplementary material 2: Fig. S2 The effects of SHI treatment on pulmonary structure. Lung tissue HE staining and Masson staining after 14 days of SHI treatment. [file 13020_2024_947_MOESM2_ESM.tif]

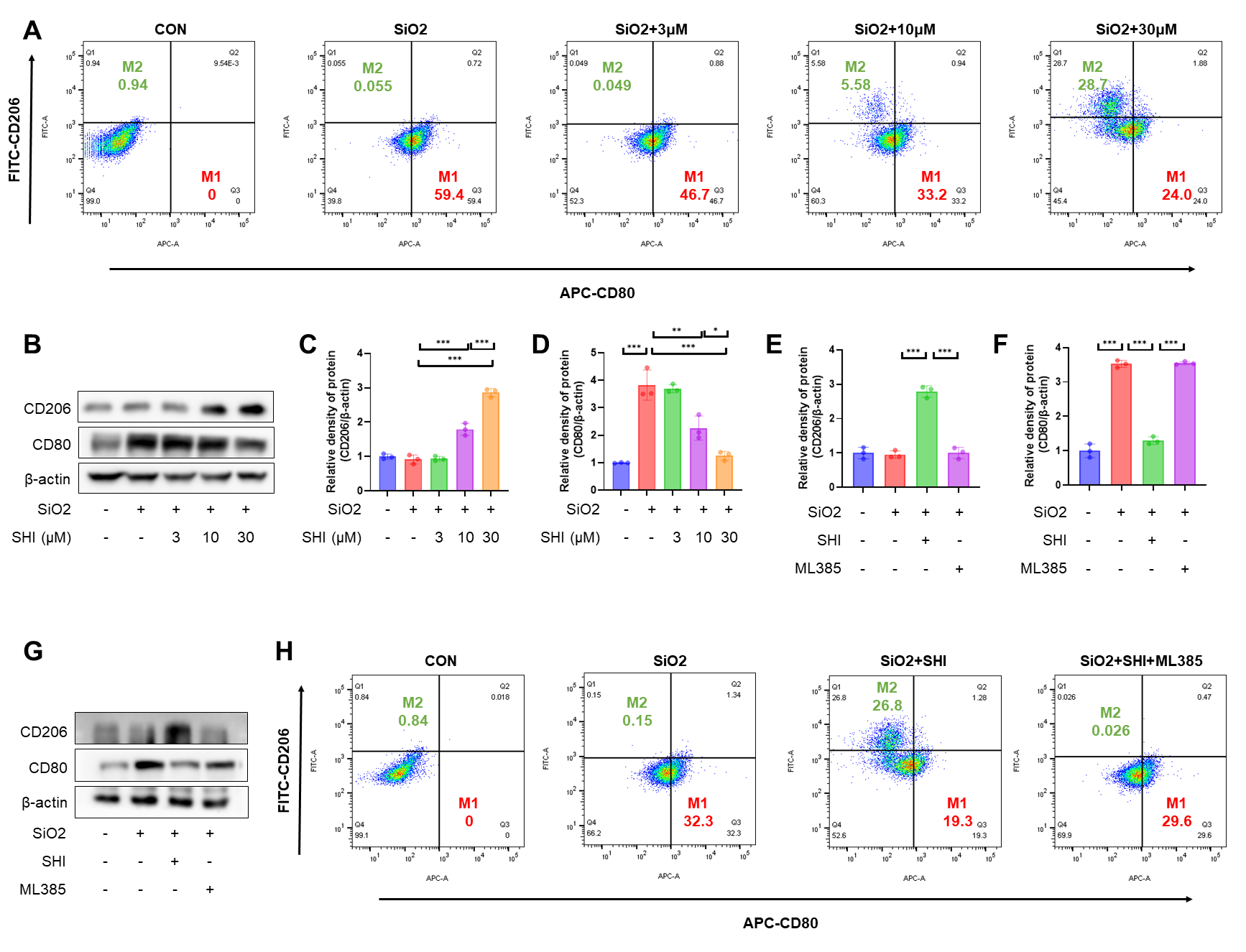

Supplement: Supplementary file 3 — Supplementary material 3: Fig. S3 The effects of SHI on macrophage polarization. (A) Flow cytometry was employed to detect macrophage polarization. APC-F4/80 was used to detect M1 polarization, while FITC-CD206 was utilized for detecting M2 polarization. (B-D) Western blotting was used to detect macrophage polarization. (E-G) Under Nrf2 treatment, Western blotting was employed to detect macrophage polarization. (H) Under Nrf2 treatment, Flow cytometry was employed to detect macrophage polarization. Data are presented as means ± standard deviation (S.D), and all experiments were independently repeated at least three times. (*P < 0.05, **P < 0.01, and ***P < 0.001). [file 13020_2024_947_MOESM3_ESM.tif]

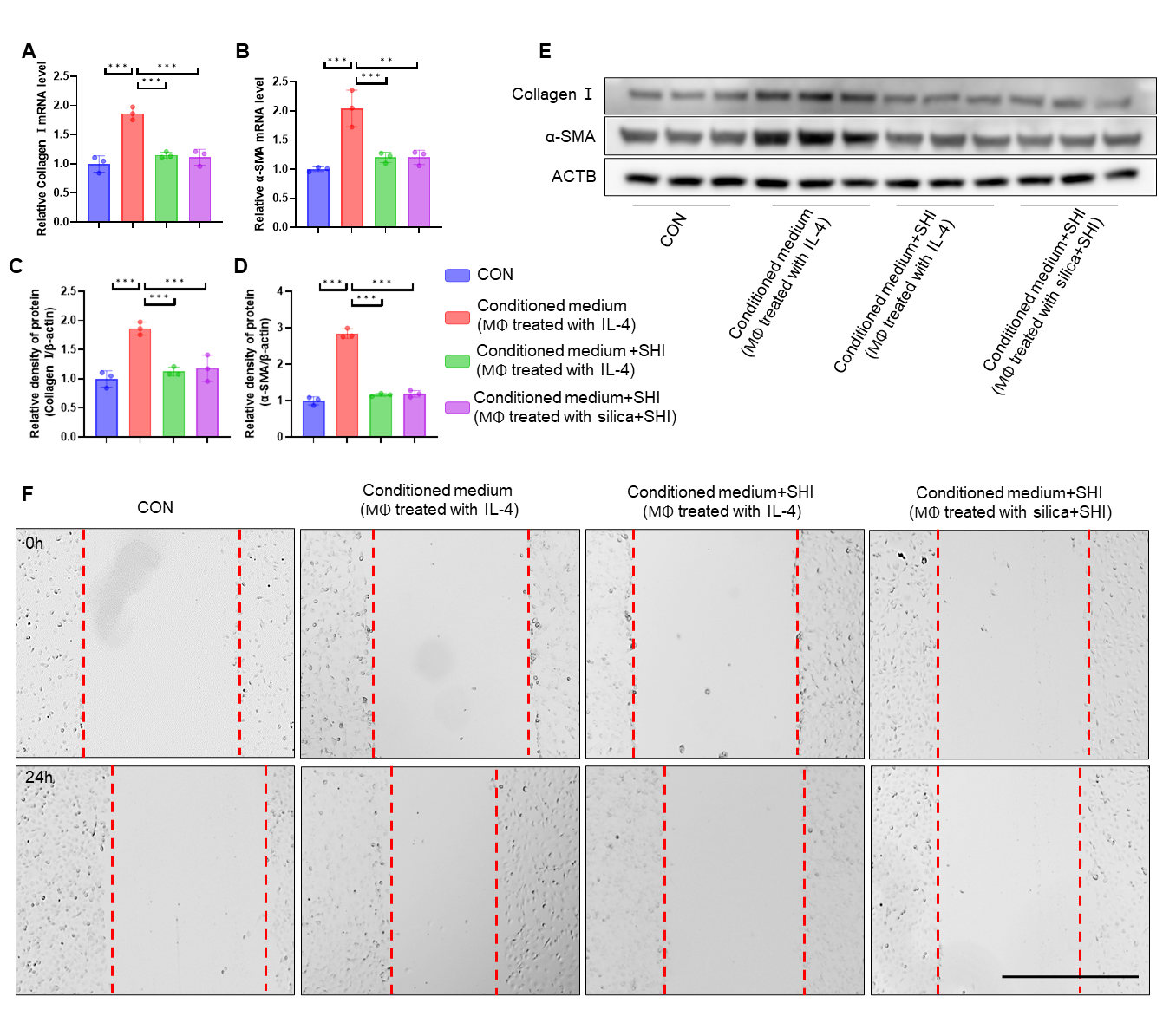

Supplement: Supplementary file 4 — Supplementary material 4: Fig. S4 The inhibitory effect of SHI on M2 macrophage-mediated activation of fibroblasts. Macrophages were treated with IL-4/Silica+SHI, and conditioned medium was obtained. The activation of fibroblasts was observed in the presence or absence of SHI. (A-B) qPCR experiments to detect mRNA levels of collagen I and α-SMA. (C-E) WB experiments to measure the expression levels of collagen I and α-SMA. (F) Scratch assay. The scale bars represent 1000 μm. Data are presented as means ± standard deviation (S.D), and all experiments were independently repeated at least three times. (*P < 0.05, **P < 0.01, and ***P < 0.001). [file 13020_2024_947_MOESM4_ESM.tif]
